# An eDNA‐based assessment of *Garra cambodgiensis* (stonelapping minnow) distribution on a megadiverse river, the Mekong

**DOI:** 10.1002/ece3.10898

**Published:** 2024-02-07

**Authors:** Maslin Osathanunkul, Chatmongkon Suwannapoom

**Affiliations:** ^1^ Department of Biology, Faculty of Science Chiang Mai University Chiang Mai Thailand; ^2^ School of Agriculture and Natural Resources University of Phayao Phayao Thailand

**Keywords:** data‐poor area, economically important fish, population decline, species occurrence and distribution, species‐specific detection, the Mekong River

## Abstract

*Garra cambodgiensis* (stonelapping minnow) has experienced significant population declines, prompting intensive research and management, although its distribution in river systems such as the Mekong remains obscure. Effective conservation and management necessitate accurate monitoring and survey data on the distribution of freshwater species. Traditional surveying techniques for fish may be challenging and generate insufficient data on species distribution. This study developed an eDNA‐based method for detecting *G. cambodgiensis* to address this void. Twenty‐one locations were surveyed. Water samples were collected in triplicate from the river's surface at each site and processed within 48 h in a dedicated laboratory. Primers and probes for *G. cambodgiensis* were meticulously designed and species‐specificity tested to ensure accurate detection without interference from co‐occurring species in the same geographic range. Each water sample was analysed by qPCR using six technical replicates. The results of qPCR were reported as positive with quantifiable eDNA concentration (copies/mL), below the limit of quantification, or non‐detectable. *G. cambodgiensis* eDNA was detected in water samples collected from 10 out of 21 sampling sites, with concentrations ranging from 8.5 to 2990.0 copies/mL. Importantly, *G. cambodgiensis* eDNA was consistently detected in all three replicate water samples at each site where the qPCR experiment yielded positive results. The findings of this study demonstrate the feasibility and effectiveness of incorporating eDNA‐based monitoring or surveys for *G. cambodgiensis* in the ecologically diverse Mekong River. Monitoring based on eDNA can aid in targeting and informing conservation and management of *G. cambodgiensis* in its natural habitat. Comprehensive and robust information on species distribution can be obtained via an eDNA‐based survey, which could contribute to more efficient and informed decision‐making processes in fisheries management and conservation efforts.

## Graphical abstract

This study developed an eDNA‐based method to accurately monitor the distribution of *Garra cambodgiensis* in the Mekong River, where population declines have raised conservation concerns. The eDNA technique successfully detected *G. cambodgiensis* in water samples from 10 out of 21 survey sites, demonstrating its feasibility and effectiveness in the ecologically diverse Mekong River. This innovative approach can enhance conservation and management efforts by providing comprehensive data on species distribution, informing decision‐making processes for fisheries management and conservation initiatives.

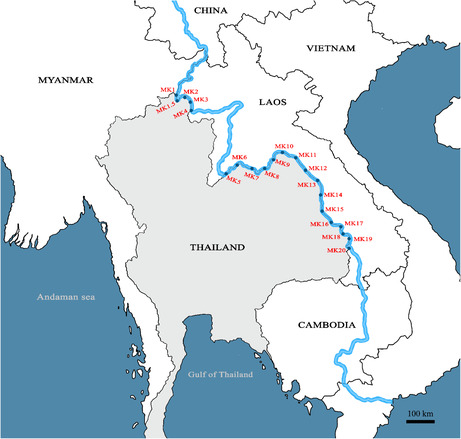

## 1 INTRODUCTION

*Garra* is a genus of freshwater fish that inhabits different regions of Asia, such as Thailand. There are seven known species of Garra in Thailand, including *Garra cambodgiensis* (Tirant 1884). The species are commonly located in fluvial and lotic environments, with occasional sightings in limnetic habitats such as ponds and reservoirs. *G. cambodgiensis*, commonly known as the stonelapping minnow, is highly valued in the commercial market and is considered a local specialty product in Northern Thailand (Pornsopin et al., 2011). The flavour of the fish, especially the females with their intact roe, is widely recognised and highly esteemed in the northern region of Thailand, and thus the fish turned out to be excessively fished for food. *G. cambodgiensis* is an aquatic species that found inhabit the Mekong River basin, spanning multiple Southeast Asian nations such as Cambodia, Laos, Thailand and Vietnam. The species is predominantly located in the upper and middle sections of the Mekong River, specifically in the Nam Ou and Nam Khan rivers in Laos, as well as the Ma River in Vietnam (Vidthayanon, 2002). The current distribution of *G. cambodgiensis* is incompletely determined and dependent on local reports, some of which have not been confirmed. A significant portion of the data in Thailand is sourced from the Department of Fisheries. However, these reports are not current, mostly because of the substantial expenses and workforce required for sampling over extensive time periods and geographical areas. *G. cambodgiensis* is distributed across many segments of the Mekong River in Thailand, namely the upper Mekong River in Chiang Rai province, the middle Mekong River in Nong Khai province and the lower Mekong River in Mukdahan and Nakhon Phanom provinces (Kottelat, 2013). However, a variety of factors, such as habitat deterioration, dam construction and excessive fishing, could affect the dispersal of *G. cambodgiensis*. An instance of this is the correlation between the construction of the Nam Ngum Dam in Laos and the decrease in populations of *G. cambodgiensis* in the Nam Ngum River. This river is a significant tributary of the Mekong River in Laos (Kottelat, 2016). In addition, the fish population is being overly taken for eating because of the highly esteemed taste of fish, especially the females with their intact roe. Concerted endeavours are underway to preserve and oversee populations of *G. cambodgiensis* in the Mekong River basin, primarily through habitat restoration and the implementation of fisheries rules. In June 2021, the Fisheries Department selected *G. cambodgiensis* as one of the 39 fish species to be included in the initiative aimed at restoring and protecting rare, endemic and endangered species. The estimation of species distribution and relative abundance is of utmost importance for fundamental ecological research and environmental evaluation (Cao et al., 2001; Kennard et al., 2006).

Traditional sample techniques, such as electrofishing, seining and gill netting, remain the primary methods for surveys and monitoring of most freshwater species (Sandstrom et al., 2013). Nevertheless, the constraints of these methods hinder the gathering of precise and adequate distribution data. Therefore, it is necessary to develop an alternate fish survey technique that is more efficient and cost‐effective while maintaining its effectiveness and reliability. Environmental DNA (eDNA) detection is a contemporary method used to monitor species by analysing genetic material found in environmental samples such as water, soil or air (Boothroyd et al., 2016; Clare et al., 2021; Deiner et al., 2017; Edwards et al., 2018; Lynggaard et al., 2022; Osathanunkul, 2019). The technique can detect the existence of a target species without the need to physically capture it. The eDNA‐based method has been shown to be a very good, quick and useful way to find aquatic organisms, especially ones that are hard to find or not common (Biggs et al., 2015; Fernández et al., 2019; Hinlo et al., 2017; Katano et al., 2017; McKee et al., 2015; Rees et al., 2014; Schneider et al., 2016). The use of eDNA‐based techniques has experienced a growing trend in the detection and surveillance of fish populations in aquatic habitats. An important benefit of eDNA‐based techniques for fish detection is their ability to rapidly and cost‐effectively survey extensive areas. Additionally, these methods can yield information about the occurrence, quantity and spatial distribution of many fish species within a single sample (Deiner et al., 2017). Environmental DNA has become a powerful method for monitoring aquatic biodiversity. Numerous studies have demonstrated its effectiveness in detecting fish species in Thai lotic and lentic water (Blackman et al., 2021; Osathanunkul, 2022; Osathanunkul & Minamoto, 2021a; Osathanunkul & Suwannapoom, 2023a, 2023b; Rodpai et al., 2023). The objective of this study was to develop an eDNA‐based survey to monitor the *G. cambodgiensis* in the Mekong River, which is the largest River Basin in Thailand and a highly profitable river fishery globally. The findings will be valuable for identifying crucial habitats, implementing conservation initiatives and addressing gaps in the species' distribution data.

## 2 MATERIALS AND METHODS

### 2.1 Ethics statement

DNA samples of the *G. cambodgiensis* used in this study were extracted from the fish mucus. The *G. cambodgiensis* are not a protected species and no specific permissions were required. After swiping mucus, the fish were released back to where they were found (three individuals). This was approved by the Animal Care and Use Committee review under protocol number: 64 02 04 005.

### 2.2 Water sampling and DNA extraction

**FIGURE 1:**
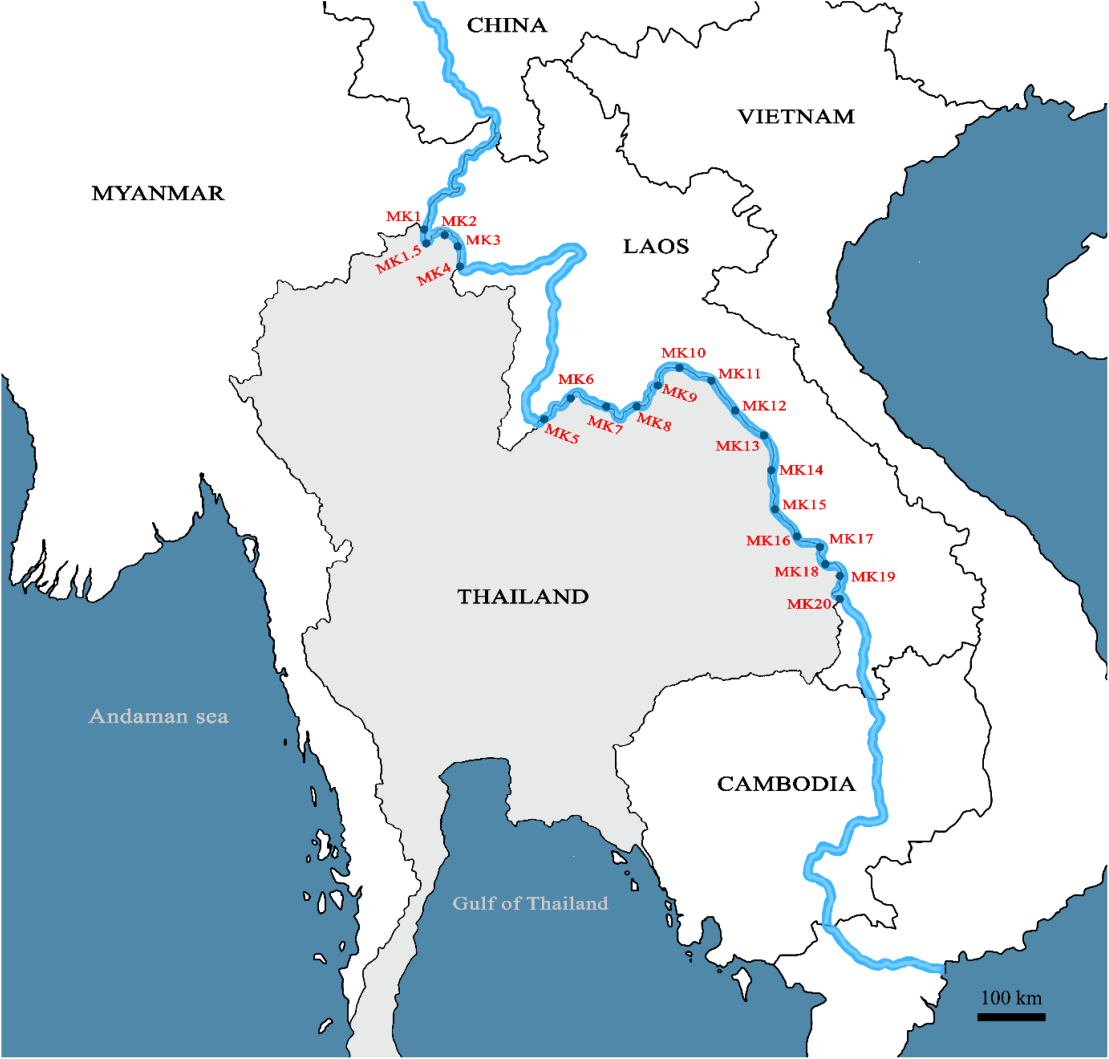
Map of 21 sampling sites located on Mekong River.

Waters samples were collected from the surface at 21 sites in the Mekong River in triplicates at each site (Figure 1). Sample collections were conducted between November and December 2021. Water sampling and DNA extraction were conducted accordingly to Osathanunkul and Minamoto (2021b). To avoid contamination, all field equipment was sterilised using 10% bleach (0.6% sodium hypochlorite), UV‐Crosslinker or autoclaved and sealed. A separate pair of nitrile disposable gloves were used for each sample. At each site, water samples were collected in a bucket that had been previously decontaminated with a 10% bleach rinse followed by two distilled water rinses. Nine hundred millilitre samples of water were immediately filtered using a BD Luer‐Lok™ syringe (sterilised with UV light for 1 h and individually sealed) with a glass fibre 0.7 μm filter (Whatman GF/F) in the field. A volume of 100 mL of water was subjected to filtration using a single filter, depending on the presence of turbidity. Consequently, a total of nine filters were employed for each individual sample. At each site, three samples were collected (*n* = 21 × 3 = 63), and each used nine filters (*N* = 63 × 9 = 567). All filters were individually stored in 1.5 mL tubes and kept in a polystyrene box containing dry ice before transferring to a −20°C freezer until extraction. Also, 900 mL of distilled water was filtered for each site as a field‐negative filtration control in the same manner as water samples. A total of nine filters were included for the extraction of eDNA from each water sample replicate. All samples were extracted within 48 h of sampling in a dedicated clean laboratory, following the DNeasy Blood and Tissue Kit (Qiagen, Hilden, Germany) method from Osathanunkul and Minamoto (2021b). Samples were eluted in 25 μL of TE buffer twice and subsequently preserved at −20°C. Each sample was then treated with the OneStep PCR Inhibitor Removal Kit (Zymo Research) to remove potential PCR inhibitors.

### 2.3 Developing qPCR assay and testing sensitivity

The specificity of the primers‐probe was first evaluated by in silico analysis using GenBank Primer‐BLAST (Ye et al., 2012). The qPCR assays were then conducted using mucus DNA to test for specificity. To minimise potential laboratory contamination, procedures for eDNA extraction from filters and qPCR assays were performed in different rooms. Working areas were bleached and sprayed with 70% EtOH, equipment and materials were also sterilised, and UV treated prior to use. Total DNA was extracted from the mucus sample from three individuals the *G. cambodgiensis* and one individual of each non‐target fish species or co‐occurring species using the Qiagen DNeasy Blood and Tissue Kit (Qiagen, Valencia, CA). Extracted DNA was used as a template for qPCR assay. Species‐specific primers and probe amplify a 109 bp targeting the mitochondrial cytochrome C oxidase subunit I gene (COI) for the *G. cambodgiensis* were used in the qPCR assays (Osathanunkul & Suwannapoom, 2023a). Sequences of the primers and probe are (Forward primer) GarraCamCOI‐F250: 5′‐GGGTTTGGAAACTGGCTC‐3′, (Reverse primer) GarraCamCOI‐R337: 5′‐ATAATAGCAGGAATGATGGTGG‐3′ and (Probe) GarraCamCOI‐P287: FAM 5′‐CCCCCGACATGGCATTTC‐3′ MGB. qPCR was conducted using species‐specific primers which were tested for species specificity with non‐target species or co‐occurring species in the same geographic range (Table 1). The alignment of the primers was conducted against all other *Garra* species in order to evaluate the presence of mismatches in the in silico test. The corresponding results can be found in Table S1.

**TABLE 1:** Species used in specificity primer test (in vitro qPCR).

Scientific name	Common name	Remark
*Garra cambodgiensis*	Stonelapping minnow	Target species
*Garra fasciacauda*	–	Closely related species
*Anabas testudineus*	Climbing perch	Co‐occurring species
*Anguilla bicolor*	Indonesian shortfin eel	Co‐occurring species
*Barbonymus gonionotus*	Silver barb	Co‐occurring species
*Channa aurolineatus*	Cobra snakehead	Co‐occurring species
*Channa micropeltes*	Giant snakehead	Co‐occurring species
*Channa striata*	Striped snakehead	Co‐occurring species
*Chitala ornata*	Clown featherback	Co‐occurring species
*Cyprinus carpio*	Common carp	Co‐occurring species
*Hypsibarbus malcolmi*	Goldfin tinfoil barb	Co‐occurring species
*Labiobarbus siamensis*	–	Co‐occurring species
*Notopterus notopterus*	Bronze featherback	Co‐occurring species
*Pangasianodon gigas*	Mekong giant catfish	Co‐occurring species
*Pangasianodon hypophthalmus*	Iridescent shark	Co‐occurring species
*Pangasius bocourti*	Basa	Co‐occurring species
*Pangasius larnaudii*	Spot pangasius	Co‐occurring species
*Probarbus jullieni*	Jullien's golden carp	Co‐occurring species
*Puntioplites proctozysron*	Smith's Barb	Co‐occurring species

The qPCR assay was deployed accordingly to Osathanunkul and Minamoto (2021b). Six technical replicates of each sample were performed. For each individual, 20 μL qPCR reaction containing 10.0 μL of 2× TaqMan Environmental Master Mix 2.0 (Thermo Fisher Scientific), 2.0 μL of DNA template, 900 nM each of the F/R primers and 125 nM of the probe were prepared in triplicate. Negative controls with all qPCR reagents but no template (three replicates) were run in parallel to monitor potential contamination. Water samples taken from the fish culture in ponds of the species were included in the qPCR assay as positive controls. The temperature gradient assay was used to optimise the qPCR conditions. The qPCR program consisted of 95°C for 10 min, followed by 50 cycles of 95°C for 15 s and 60°C for 1 min. All qPCR reactions were carried out using Rotor‐Gene Q MDx 5plex (Qiagen, Valencia, CA). qPCR of water samples was performed similarly to the mucus DNA tests (see above) with a few minor differences. A total of six qPCR reactions were performed for every group of three replicates of water samples.

### 2.4 PCR inhibition test

The inhibition of the PCRs of water samples was performed as described in a previous study (Doi et al., 2017). Primers and probes targeting the 16S rRNA of jellyfish species, *Chiropsoides buitendijki*, a marine species which does not inhabit the streams, were used to test for inhibition as internal controls (forward primer: 5′‐CCCCAATCGAAATTAAGTTAGCC‐3′; reverse primer: 5′‐CACAGGTAGAGTGGAGAAATAGAG‐3′; probe: 5′‐FAM‐GTGAAGACGCAGCTTTGTCT‐TAMRA‐3′). The oligonucleotide synthesis of *C. buitendijki* (1.5 × 10^2^ copies) was added to the samples (gBlocks™ Gene Fragments, IDT). According to Hartman et al., 2005, ΔCq or Cq shift of ≥3 cycles of the internal controls in the water sample from the internal controls in negative controls indicates inhibition.

### 2.5 Statistical analyses

#### 2.5.1 Calculation of limit of detection (LOD) and limit of quantification (LOQ)

An eight‐point standard dilution curve was made using synthetic DNA Fragment (gBlocks™ Gene Fragments, IDT) to test how well and how sensitively the assay worked. This curve was then used to compute the amounts of eDNA in the samples. The LOD and LOQ computations were executed using the R script supplied by Klymus et al. (2020), following the methodology outlined in their article. The DNA copy numbers per microlitre (copies/mL) of water from each sample were quantified using the approach outlined by Agersnap et al. (2017).

#### 2.5.2 Calculation of eDNA copy number in water samples

The concentration of each sample was calculated based on the synthesised target gene standard curve (gBlocks™ Gene Fragments, IDT) and reported as copies/mL. The copy numbers were calculated using the equation: CL = [Cr*(Ve/Vr)]/Vw. CL refers to the concentration of eDNA copies per microlitre of water in this particular situation. Cr represents the number of eDNA copies that have been identified within the given volume of the reaction. The term ‘Ve’ refers to the total volume collected as a result of the extraction procedure. Vr represents the volume of the extracted substance that is used in the qPCR reaction. Vw represents the measured volume of water that has undergone the filtration process. All qPCR results were reported into three categories which are (1) *positive* with quantifiable eDNA concentration, below limit of quantification (*bq*: Cq = 37.72–44.99) and non‐detect (*nd*: Cq ≥ 50 or No amplification).

#### 2.5.3 Evaluation of false positives and false negatives

The R package eDNAShinyApp (Diana et al., 2021) was used to assess the error rates of surveys targeting *G. cambodgiensis* eDNA detection, specifically the rates of false positives and false negatives. The package was executed with the following parameters: a probability of site occupancy of 0.5, a variance of probability of site occupancy of 4, a variance of coefficients of probability of site occupancy of 0.25 and a number of significant covariates of 2. The execution consisted of 5000 burn‐in iterations, 10,000 iterations using 1 chain and 100 thinned iterations. Essentially, the run produced probabilities of occupancy (ψ), true positive (θ_11_) and false positive (θ_10_) observations from Stage 1 (sample collection), and true positive (ρ_11_) and false positive (ρ_10_) observations from Stage 2 (laboratory analysis), as explained in reference (Diana et al., 2021).

## 3 RESULTS

### 3.1 Specificity and sensitivity of the qPCR assay

An in silico evaluation of the primer pair's specificity revealed that the primers are unique to the *G. cambodgiensis* due to a number of mismatches between the target species, closely related and co‐occurring species (Table S1). Testing of the COI qPCR assay on the DNA of *G. cambodgiensis* and DNA from non‐target fish found that the assay is highly specific to *G. cambodgiensis* DNA and demonstrated efficient amplification exclusively from the target species and no amplification from other tested fish. There was no amplification of any non‐target fish (no amplification after 45 cycles) except for one out of six qPCR replicates for *Barbonymus gonionotus* and *Notopterus notopterus*, which yielded Cq values of 50.18 and 50.72 respectively. The DNA of *G. cambodgiensis* amplified effectively (Cq values between 20 and 27) for both DNA fragment standards and mucus DNA. It was determined that both the limit of detection (LOD) and the limit of quantitation (LOQ) were 25.44 copies/reaction (2.12 copies/mL). The Cq cut‐off for positive detection was determined to be 37.71, based on the calculated LOD and LOQ.

### 3.2 Inhibition in water samples

The average of ΔCq values from the internal controls of all samples was less than 3, which is lower than the inhibition criteria (Cq shift of ≥3 cycles was an indication of inhibition) (Table 2). Therefore, PCR inhibition was not likely to occur in all samples.

**TABLE 2:** Cq values and average ∆Cq' of water samples from PCR inhibition test.

Sample	Cq	ΔCq	Sample	Cq	ΔCq	Sample	Cq	ΔCq
*C. buitendijki*	29.31	–	*C. buitendijki*	29.31	–	*C. buitendijki*	29.31	–
MK1	29.87	0.56	MK7	30.79	1.48	MK14	28.11	−1.2
MK1	29.92	0.61	MK7	30.33	1.02	MK14	28.33	−0.98
MK1	30.06	0.75	MK7	30.64	1.33	MK14	27.9	−1.41
MK1.5	30.14	0.83	MK8	28.66	−0.65	MK15	29.08	−0.23
MK1.5	29.96	0.65	MK8	29.12	−0.19	MK15	28.67	−0.64
MK1.5	29.92	0.61	MK8	28.95	−0.36	MK15	28.76	−0.55
MK2	30.24	0.93	MK9	28.81	−0.5	MK16	30.55	1.24
MK2	30.52	1.21	MK9	28.86	−0.45	MK16	30.37	1.06
MK2	30.17	0.86	MK9	29.01	−0.3	MK16	30.44	1.13
MK3	29.94	0.63	MK10	28.62	−0.69	MK17	30.26	0.95
MK3	29.98	0.67	MK10	29.27	−0.04	MK17	30.47	1.16
MK3	30.24	0.93	MK10	29.15	−0.16	MK17	30.1	0.79
MK4	29.21	−0.1	MK11	29.99	0.68	MK18	30.57	1.26
MK4	29.05	−0.26	MK11	29.73	0.42	MK18	30.48	1.17
MK4	28.9	−0.41	MK11	30.34	1.03	MK18	30.12	0.81
MK5	28.64	−0.67	MK12	28.49	−0.82	MK19	28.77	−0.54
MK5	28.76	−0.55	MK12	28.69	−0.62	MK19	28.95	−0.36
MK5	28.9	−0.41	MK12	28.38	−0.93	MK19	28.69	−0.62
MK6	29.08	−0.23	MK13	30.25	0.94	MK20	29.9	0.59
MK6	28.86	−0.45	MK13	30.33	1.02	MK20	29.68	0.37
MK6	29.2	−0.11	MK13	30.07	0.76	MK20	30.15	0.84

*Note*: Three replicates of samples collected from 21 sites on the Mekong River.

### 3.3 eDNA detection in water samples

*Garra cambodgiensis* eDNA was discovered in 100 percent of water samples collected from the pond where *G. cambodgiensis* reside (used as positive controls). The negative controls (deionised water) did not produce any DNA amplification. The spread of *G. cambodgiensis* in the Mekong River was determined by qPCR analysis of water samples from 21 sampling locations. For the 63 samples ascribed to the 2021 water sample collection, a total of 189 qPCR reactions were performed. In 10 sampling locations (MK4–MK6, MK8–MK10, MK12, MK14–MK15 and MK19), *G. cambodgiensis* eDNA was identified (Table 3). MK14 water samples contained the highest concentration of *G. cambodgiensis* eDNA (2990.0 copies/mL), while MK9 water samples contained the lowest concentration (8.5 copies/mL). *G. cambodgiensis* eDNA was detected in all three replicates of water samples from each site where the qPCR assay was positive. There was no detectable eDNA (nq) of any *G. cambodgiensis* in water samples from six sampling sites (MK2, MK7, MK13 and MK16–MK18). Five sampling locations have qPCR findings below the limit of quantification (bq).

**TABLE 3:** qPCR results reported into three categories which are (1) positive with quantifiable eDNA concentration (copies/mL), (2) below limit of quantification (bq: Cq = 37.72–44.99) and (3) non‐detect (nd: Cq ≥ 45 or No amplification).

Site	Province	Cq (average)	eDNA concentration (copie/mL)	Biological replicates^a^	Technical replicates^b^
MK1	Chiang Rai^c^	38.50	bq	0/3	2/6
MK1.5		37.91	bq	1/3	2/6
MK2		49.19	nd	0/3	0/6
MK3		38.59	bq	0/3	1/6
MK4		31.72	95.3	3/3	6/6
MK5	Loei	28.75	626.1	3/3	6/6
MK6		30.15	257.3	3/3	6/6
MK07	Nong Khai^c^	49.26	nd	0/3	0/6
MK08		29.71	341.6	3/3	6/6
MK09		35.53	8.5	3/3	6/6
MK10	Bueng Kan	28.96	550.1	3/3	6/6
MK11		38.02	bq	0/3	2/6
MK12	Nakhon Phanom^c^	26.68	2329.3	3/3	6/6
MK13		49.29	nd	0/3	0/6
MK14		26.29	2990.0	3/3	6/6
MK15	Mukdahan^c^	27.47	1413.5	3/3	6/6
MK16	Amnat charoen	–	nd	0/3	0/6
MK17	Ubon Ratchathani	48.74	nd	0/3	0/6
MK18		–	nd	0/3	0/6
MK19		30.91	159.1	3/3	6/6
MK20		44.76	bq	0/3	0/6

^a^ Values are the number of positive water samples out of the total number of collected samples.

^b^ qPCR replicates positive for the *G. cambodgiensis*.

^c^ Province where there were reports of *G. cambodgiensis*.

### 3.4 False positives and false negatives of the eDNA detection

The average value of the posterior mean occupancy for all locations was 0.477, with a 95% posterior credible interval (PCI) ranging from 0.959 to 1.000 (Table 4). The rate of false positive results was found to be very low through both Stage 1, which involves sample collection (θ_10_: 0.101; PCI = 0.117 to 0.274), and Stage 2, which involves laboratory analysis (p_10_: 0.101; PCI = 0.119 to 0.205). On the other hand, the rate of true positive results was high during both Stage 1 (θ_11_: 0.899; PCI = 0.676 to 0.913) and Stage 2 (p_11_: 0.899; PCI = 0.900 to 0.914).

**TABLE 4:** Posterior summaries of the probabilities of a positive observation, true or false, in both stages of the *G. cambodgiensis* survey.

Probability of	Parameter	Mean (95% PCI)
Species presence	Occurrence (ψ)	0.477 (0.959, 1.000)
True positives	Field (θ_11_)	0.899 (0.676, 0.913)
	Lab (p_11_)	0.899 (0.900, 0.914)
False positives	Field (θ_10_)	0.101 (0.117, 0.274)
	Lab (p_10_)	0.101 (0.119, 0.205)

The analysis of conditional probability of species absence reveals a significant likelihood of species absence when 0 to 2 replicates are amplified, followed by a decrease in likelihood when three replicates are amplified. Conversely, there is a low likelihood of species absence when samples are amplified with 4 to 6 replicates (refer to the first row of Table 5). The posterior probability of zero qPCR positives given species presence is only 5%, and this likelihood reduces for *x* = 1, 2, 3, 4 before it begins to climb, reaching nearly 50% at *x* = 6 (refer to the second row of Table 5).

**TABLE 5:** The posterior conditional probabilities of species absence (1 − ψ(*x*)), given *x* (0–6) amplifying qPCR replicates and posterior probability of *x* (0–6) positive qPCR replicated conditional on species presence.

*x*	0	1	2	3	4	5	6
1 − ψ(*x*)	0.89533146	0.89520742	0.88604566	0.56383050	0.16695486	0.15290999	0.15271953
q(*x*)	0.05187615	0.03602515	0.01164645	0.01519825	0.08978202	0.31885230	0.47661969

## 4 DISCUSSION

*Garra cambodgiensis* faces many threats, including habitat destruction from dam building to overcollection for food, and so the population of them has undergone a significant drop. To develop an effective conservation plan or management strategy, having species distribution data is crucial. However, there is an absence of distribution data for *G. cambodgiensis*. The lack of distribution or presence of *G. cambodgiensis* in Thailand can be attributed to financial limitations and the use of unreliable monitoring methods. Mekong River is the most utilised river in the region for fisheries and irrigation. Monitoring the amount and distribution of economically or ecologically significant fish is essential for the rational, efficient and sustainable management of fisheries. Existing data on *G. cambodgiensis* occurrence and distribution in Thailand are scant, with only a handful of outdated or region‐specific publications (Kulabtong & Mahaprom, 2016; Lothongkham, 2008; Rainboth, 1996). Employing eDNA‐based detection methodologies can reduce the limitations of conventional methods, including the detection bias that can result in the non‐detection of target animals even when they are present (Simpfendorfer et al., 2016). Environmental DNA analysis additionally involves less sample effort and can be less expensive than triple‐pass electrofishing (Evans et al., 2017). In addition, with endangered or elusive species, eDNA‐based detection can be highly effective in mitigating environmental and species‐specific harm (Belle et al., 2019).

In this investigation, eDNA detection was shown to be positive at sampling locations in Chiang Rai (site MK4), Nong Khai (sites MK8 and MK9), Nakhon Phanom (sites MK12 and MK14) and Mukdahan (site MK15). Noted, *G. cambodgiensis* eDNA was only discovered at site MK4, despite the fact that sites MK1–MK4 are all located in Chiang Rai province. Also, site MK7–MK9 in Nong Khai province and site MK12–MK14 in Nakhon Phanom province, not all of them had a positive eDNA signal. The occurrence of *G. cambodgiensis* in shallow river areas with stony substrates and dense vegetation is most likely due to the habitat preference of the species, as described above (Kottelat, 2013). There is a possibility that sampling sites containing no eDNA of *G. cambodgiensis* in provinces with records of the species' presence are not the species' preferred environment. Our results suggested that sampling locations should be selected with care, taking into account the environmental preferences of the target species and their distribution. In addition, in situ validation of the eDNA data should be conducted by surveying areas with and without the target species (Thalinger et al., 2021). However, conducting fish surveys in large, species‐rich rivers such as the Mekong usually necessitates a significant expenditure of time, finances and manpower. Hence, it would be a challenging endeavour. Nevertheless, it seems that eDNA can be used to deduce the existence of *G. cambodgiensis* in the Mekong with reduced financial, temporal and labour investments, as evidenced by the outcomes of the analysis on false and true positives conducted in R, according to Diana et al. (2021). This work showcases the efficacy of employing eDNA to detect *G. cambodgiensis* in Mekong River. The occurrence of false positive results was small for both field and laboratory testing, but the probability of true positive results was significant. Therefore, the eDNA‐based detection method established in this study would undeniably be valuable for generating distribution data, documenting occurrences and creating range maps for *G. cambodgiensis* in the Mekong River.

## AUTHOR CONTRIBUTIONS

**Maslin Osathanunkul:** Conceptualization (lead); data curation (lead); formal analysis (lead); funding acquisition (lead); investigation (lead); methodology (lead); resources (lead); validation (equal); visualization (equal); writing – original draft (lead); writing – review and editing (equal). **Chatmongkon Suwannapoom:** Conceptualization (supporting); data curation (supporting); formal analysis (supporting); investigation (supporting); methodology (supporting); resources (supporting); validation (equal); visualization (equal); writing – original draft (supporting); writing – review and editing (equal).

## CONFLICT OF INTEREST STATEMENT

The authors have no conflicts of interest to declare.

## Supporting information


Table S1.

Table S2.

## Data Availability

Data necessary to replicate this study are in Table S2.
