# Supplementary material for: An eDNA‐based assessment of Garra cambodgiensis (stonelapping minnow) distribution on a megadiverse river, the Mekong
Source: Ecol Evol. 2024 Feb 7;14(2):e10898. doi: 10.1002/ece3.10898 (PMC10850809; doi:10.1002/ece3.10898)
Supplement: Supplementary file 1 — Table S1. [file ECE3-14-e10898-s001.docx]

**Supplementary Table 1**. Number of mismatches from the target species to the closely related species from alignment analysis.

| **No.** | **Accession number** | **Species** | **Forward primer** | | | | | | | | | | | | | | | | | | **Reverse primer** | | | | | | | | | | | | | | | | | | | | | |
| --- | --- | --- | --- | --- | --- | --- | --- | --- | --- | --- | --- | --- | --- | --- | --- | --- | --- | --- | --- | --- | --- | --- | --- | --- | --- | --- | --- | --- | --- | --- | --- | --- | --- | --- | --- | --- | --- | --- | --- | --- | --- | --- |
| * | MN342588 | *Garra cambodgiensis** | G | G | G | T | T | T | G | G | A | A | A | C | T | G | G | C | T | C | C | C | A | C | C | A | T | C | A | T | T | C | C | T | G | C | T | A | T | T | A | T |
| 1 | MN254983 | *Garra amirhosseini* | . | . | . | . | . | . | . | . | G | . | . | T | . | . | A | . | . | T | . | . | C | . | . | . | . | . | . | . | . | T | . | . | A | T | . | . | C | . | . | . |
| 2 | MK599486 | *Garra annandalei* | . | . | A | . | . | . | . | . | . | . | . | . | . | . | . | . | . | . | . | . | C | . | . | . | . | . | . | . | . | . | . | . | . | . | . | . | C | . | . | . |
| 3 | KF511539 | *Garra arupi* | . | . | A | . | . | . | . | . | . | . | . | . | . | . | A | . | . | . | . | . | C | . | . | . | . | . | . | . | . | . | . | . | A | T | . | G | . | . | . | C |
| 4 | KM214738 | *Garra barreimiae* | . | . | A | . | . | . | . | . | G | . | . | T | . | . | A | . | . | T | . | . | C | . | . | . | . | . | G | . | . | T | . | . | A | T | . | . | . | . | . | . |
| 5 | MK440698 | *Garra bicornuta* | . | . | A | . | . | . | . | . | . | . | . | . | . | . | A | . | . | . | . | . | T | . | . | . | . | . | . | . | . | . | . | . | . | . | . | . | . | . | . | . |
| 6 | JQ864615 | *Garra bispinosa* | . | . | . | . | . | . | . | . | . | . | . | . | . | . | A | . | . | . | . | . | C | . | . | G | . | . | . | . | . | . | . | . | A | T | . | G | . | . | . | . |
| 7 | KT799816 | *Garra borneensis* | . | . | . | . | . | . | . | . | . | . | . | . | . | . | A | . | . | . | . | . | C | . | . | . | . | . | . | . | . | T | . | . | A | T | . | . | C | . | . | C |
| 8 | KM214723 | *Garra buettikerii* | . | . | . | . | . | . | . | . | G | . | . | . | . | . | A | . | . | . | . | . | C | . | . | . | . | . | . | . | . | T | . | . | . | T | . | . | C | . | . | C |
| 9 | JF915605 | *Garra ceylonensis* | . | . | A | . | . | C | . | . | . | . | . | . | . | . | A | . | . | . | . | . | C | . | . | . | . | . | . | . | . | T | . | . | T | . | . | . | C | . | . | . |
| 10 | HM418168 | *Garra congoensis* | . | . | . | . | . | . | . | . | . | . | . | . | . | . | A | . | . | T | . | . | C | . | . | . | . | . | . | . | . | T | . | . | A | T | . | . | C | . | . | . |
| 11 | KM610626 | *Garra cryptonemus* | . | . | A | . | . | C | . | . | . | . | . | . | . | . | A | . | . | . | . | . | C | . | . | C | . | . | . | . | . | . | . | . | . | . | . | . | C | . | C | C |
| 12 | MK572208 | *Garra dampaensis* | . | . | A | . | . | . | . | . | . | . | . | . | . | . | A | . | . | T | . | . | C | . | . | . | . | . | . | . | . | T | T | . | A | . | . | . | . | . | . | . |
| 13 | KP069476 | *Garra dunsirei* | . | . | . | . | . | . | . | . | G | . | . | . | . | . | A | . | . | . | . | . | C | . | . | . | . | . | . | . | . | T | . | . | . | T | . | . | C | . | . | C |
| 14 | KM214721 | *Garra elegans* | . | . | . | . | . | . | . | . | G | . | . | T | . | . | A | . | . | T | . | . | C | . | . | . | . | . | . | . | . | T | . | . | A | T | . | . | C | . | . | . |
| 15 | JQ864618 | *Garra fasciacauda** | . | . | . | . | . | C | . | . | . | . | . | . | . | . | A | . | . | T | . | . | C | . | . | . | . | . | . | . | . | T | . | . | A | . | . | . | C | . | . | C |
| 16 | JQ864619 | *Garra findolabium* | . | . | . | . | . | . | . | . | G | . | . | . | . | . | A | . | . | T | . | . | C | . | . | . | . | . | G | . | . | . | . | . | . | . | . | . | C | . | T | C |
| 17 | MN342590 | *Garra flavatra* | . | . | A | . | . | . | . | . | . | . | . | . | . | . | A | . | . | . | . | . | C | . | . | . | . | . | . | . | . | T | T | . | A | T | . | . | . | . | . | . |
| 18 | MK902685 | *Garra fuliginosa** | . | . | . | . | . | . | . | . | . | . | . | . | . | . | A | . | . | . | . | . | C | . | . | . | . | . | . | . | . | . | . | . | A | T | . | . | C | . | . | . |
| 19 | MK599505 | *Garra gotyla* | . | . | . | . | . | . | . | . | . | . | . | . | . | . | A | . | . | . | . | . | C | . | . | . | . | . | . | . | . | . | . | . | A | T | . | . | . | . | . | . |
| 20 | KM214788 | *Garra ghorensis* | . | . | . | . | . | . | . | . | . | . | . | T | . | . | A | . | . | T | . | . | C | . | . | G | . | . | . | . | . | T | T | . | A | T | . | . | . | . | . | C |
| 21 | MN342595 | *Garra gravelyi* | . | . | . | . | . | . | . | . | . | . | . | T | . | . | A | . | . | . | . | . | C | . | . | G | . | . | G | . | . | . | . | . | A | T | . | . | . | . | . | . |
| 22 | KX570881 | *Garra gymnothorax* | . | . | . | . | . | . | . | . | . | . | . | T | . | . | A | . | . | T | . | . | C | . | . | . | . | . | . | . | . | T | . | . | A | . | . | . | C | . | . | . |
| 23 | JQ864621 | *Garra hainanensis* | . | . | A | . | . | . | . | . | . | . | . | . | . | . | A | . | . | T | . | . | C | . | . | . | . | . | . | . | . | . | . | . | . | . | . | . | C | . | C | C |
| 24 | KX946633 | *Garra hughi* | . | . | A | . | . | C | . | . | . | . | . | . | . | . | A | . | . | . | . | . | C | . | . | . | . | . | . | . | . | . | . | . | C | . | . | . | C | . | . | . |
| 25 | MN167170 | *Garra jamila* | . | . | A | . | . | . | . | . | . | . | . | . | . | . | A | . | . | T | . | . | C | . | . | . | . | . | . | . | . | . | . | . | A | T | . | . | C | . | . | C |
| 26 | JQ864620 | *Garra imberba* | . | . | A | . | . | . | . | . | . | . | . | . | . | . | A | . | . | T | . | . | C | . | . | . | . | . | . | . | . | . | . | . | . | . | . | . | C | . | C | C |
| 27 | MN167169 | *Garra jamila* | . | . | A | . | . | . | . | . | . | . | . | . | . | . | A | . | . | T | . | . | C | . | . | . | . | . | . | . | . | . | . | . | A | T | . | . | C | . | . | C |
| 28 | KM214710 | *Garra jordanica* | . | . | . | . | . | . | . | . | G | . | . | T | . | . | A | . | . | T | . | . | C | . | . | G | . | . | . | . | . | T | . | . | A | T | . | . | . | . | . | C |
| 29 | MK599483 | *Garra kempi* | . | . | . | . | . | . | . | . | . | . | . | . | . | . | A | . | . | . | . | . | C | . | . | . | . | . | . | . | . | T | . | . | T | . | . | . | C | . | G | C |
| 30 | MK572210 | *Garra lamta* | . | . | . | . | . | . | . | . | . | . | . | . | . | . | A | . | . | . | . | . | C | . | . | . | . | . | C | . | . | . | . | . | A | . | . | . | C | . | . | . |
| 31 | MK277208 | *Garra lissorhynchus* | . | . | A | . | . | C | . | . | . | . | . | . | . | . | A | . | . | T | . | . | C | . | . | . | . | . | . | . | . | T | . | . | A | . | . | G | . | . | . | . |
| 32 | KM214705 | *Garra longipinnis* | . | . | . | . | . | . | . | . | . | . | . | T | . | . | A | . | . | A | . | . | C | . | . | . | . | . | G | . | . | T | . | . | A | T | . | . | C | . | . | . |
| 33 | MG852060 | *Garra lorestanensis* | . | . | . | . | . | . | . | . | . | . | . | T | . | . | A | . | . | T | . | . | C | . | . | . | . | . | . | C | . | T | . | . | A | . | . | . | C | . | . | . |
| 34 | KX239495 | *Garra mcclellandi* | . | . | A | . | . | C | . | . | . | . | . | . | . | . | A | . | . | . | . | . | C | . | . | . | . | . | . | . | . | . | . | . | C | . | . | . | C | . | . | . |
| 35 | GU086605 | *Garra micropulvinus* | . | . | A | . | . | . | . | . | . | . | . | . | . | . | A | . | . | T | . | . | C | . | . | . | . | . | . | . | . | . | . | . | . | . | . | . | C | . | C | C |
| 36 | KT223106 | *Garra mini* | . | . | C | . | . | C | . | . | . | . | . | . | . | . | A | . | . | . | . | . | . | . | . | . | . | . | . | . | . | T | T | . | A | . | . | T | C | . | G | C |
| 37 | JQ864605 | *Garra mirofrontis* | . | . | C | . | . | . | . | . | C | . | . | . | . | . | A | . | . | A | . | . | . | . | . | . | . | . | T | . | . | . | . | . | A | . | . | . | C | . | T | C |
| 38 | MG852030 | *Garra mondica* | . | . | . | . | . | . | . | . | G | . | . | T | . | . | A | . | . | T | . | . | C | . | . | . | . | . | . | . | . | T | . | . | A | T | . | . | C | . | . | . |
| 39 | JX983296 | *Garra mullya* | . | . | A | . | . | . | . | . | . | . | . | . | . | . | A | . | . | . | . | . | C | . | . | . | . | . | . | . | . | . | . | . | C | . | . | . | C | . | . | . |
| 40 | KX951813 | *Garra naganensis* | . | . | A | . | . | . | . | . | . | . | . | . | . | . | A | . | . | . | . | . | C | . | . | . | . | . | C | . | . | . | . | . | A | . | . | . | C | . | . |  |

*Species that found in Mekong River

**Supplementary Table 1**. (cont.) Number of mismatches from the target species to the closely related species from alignment analysis.

| **No.** | **Accession number** | **Species** | **Forward primer** | | | | | | | | | | | | | | | | | | **Reverse primer** | | | | | | | | | | | | | | | | | | | | | |
| --- | --- | --- | --- | --- | --- | --- | --- | --- | --- | --- | --- | --- | --- | --- | --- | --- | --- | --- | --- | --- | --- | --- | --- | --- | --- | --- | --- | --- | --- | --- | --- | --- | --- | --- | --- | --- | --- | --- | --- | --- | --- | --- |
| * | MN342588 | *Garra cambodgiensis** | G | G | G | T | T | T | G | G | A | A | A | C | T | G | G | C | T | C | C | C | A | C | C | A | T | C | A | T | T | C | C | T | G | C | T | A | T | T | A | T |
| 41 | KM214757 | *Garra nana* | . | . | . | . | . | . | . | . | . | . | . | . | . | . | A | . | . | . | . | . | T | . | . | G | . | . | . | . | . | T | . | . | A | T | . | . | C | . | . | . |
| 42 | MN167173 | *Garra napata* | . | . | A | . | . | . | . | . | . | . | . | . | . | . | A | . | . | T | . | . | C | . | . | . | . | . | . | . | . | . | . | . | A | T | . | . | C | . | . | C |
| 43 | MN563573 | *Garra nasuta* | . | . | . | . | . | . | . | . | . | . | . | . | . | . | A | . | . | . | . | . | C | . | . | . | . | . | . | . | . | . | . | . | A | . | . | . | . | . | . | . |
| 44 | MN258739 | *Garra nudiventris* | . | . | A | . | . | . | . | . | . | . | . | . | . | . | A | . | . | . | . | . | C | . | . | . | . | . | . | . | . | T | . | . | . | . | . | T | C | . | G | . |
| 45 | MT884554 | *Garra orientalis* | . | . | . | . | . | . | . | . | . | . | . | . | . | . | A | . | . | . | . | . | C | . | . | G | . | . | . | . | . | . | . | . | A | T | . | . | . | . | . | . |
| 46 | MK074286 | *Garra ornata* | . | . | A | . | . | . | . | . | . | . | . | . | . | . | A | . | . | T | . | . | C | . | . | . | . | . | T | . | . | T | . | . | A | T | . | . | C | . | . | . |
| 47 | KJ909429 | *Garra paralissorhynchus* | . | . | A | . | . | C | . | . | . | . | . | . | . | . | A | . | . | T | . | . | C | . | . | . | . | . | . | . | . | . | . | . | A | . | . | . | . | . | G | . |
| 48 | MN254987 | *Garra persica* | . | . | . | . | . | . | . | . | G | . | . | T | . | . | A | . | . | T | . | . | C | . | . | . | . | . | . | . | . | T | . | . | A | T | . | . | C | . | . | . |
| 49 | JQ864604 | *Garra qiaojiensis* | . | . | . | . | . | . | . | . | . | . | . | . | . | . | A | . | . | . | . | . | C | . | . | . | . | . | . | . | . | . | . | . | A | T | . | . | . | . | . | . |
| 50 | MN832847 | *Garra quadratirostris* | . | . | A | . | . | . | . | . | . | . | . | . | . | . | A | . | . | . | . | . | C | . | . | . | . | . | . | . | . | . | . | . | . | T | . | . | . | . | . | . |
| 51 | KX399142 | *Garra rakhinica* | . | . | A | . | . | . | . | . | G | . | . | . | . | . | A | . | . | T | . | . | C | . | . | . | . | . | . | . | . | T | T | . | A | . | . | T | C | . | . | . |
| 52 | MN258738 | *Garra roseae* | . | . | A | . | . | . | . | . | . | . | . | . | . | . | A | . | . | T | . | . | C | . | . | . | . | . | . | . | . | T | . | . | . | . | . | T | C | . | . | . |
| 53 | KM214726 | *Garra rossica* | . | . | A | . | . | . | . | . | . | . | . | . | . | . | A | . | . | . | . | . | C | . | . | . | . | . | . | . | . | T | . | . | . | . | . | T | C | . | G | . |
| 54 | JQ864616 | *Garra rotundinasus* | . | . | A | . | . | . | . | . | . | . | . | . | . | . | A | . | . | T | . | . | C | . | . | . | . | . | . | . | . | . | . | . | T | . | . | . | C | . | C | C |
| 55 | MN342597 | *Garra rufa* | . | . | . | . | . | . | . | . | G | . | . | T | . | . | A | . | . | T | . | . | C | . | . | . | . | . | . | . | . | T | . | . | A | T | . | . | . | . | . | C |
| 56 | KM214718 | *Garra sahilia* | . | . | A | . | . | . | . | . | . | . | . | T | . | . | A | . | . | T | . | . | C | . | . | . | . | . | . | . | . | T | . | . | A | T | . | . | C | . | . | . |
| 57 | MK902688 | *Garra salweenica* | . | . | . | . | . | . | . | . | . | . | . | . | . | . | A | . | . | . | . | . | C | . | . | . | . | . | . | . | . | . | . | . | A | . | . | . | C | . | . | . |
| 58 | MN167174 | *Garra sannarensis* | . | . | . | . | . | . | . | . | . | . | . | . | . | . | A | . | . | T | . | . | C | . | . | . | . | . | . | . | . | . | . | . | A | T | . | . | C | . | . | C |
| 59 | KM214744 | *Garra smarti* | . | . | . | . | . | . | . | . | G | . | . | . | . | . | A | . | . | . | . | . | C | . | . | . | . | . | . | . | . | T | . | . | . | T | . | . | C | . | . | C |
| 60 | KX983933 | *Garra surgifrons* | . | . | . | . | . | . | . | . | . | . | . | . | . | . | A | . | . | . | . | . | C | . | . | . | . | . | . | . | . | . | . | . | A | . | . | . | C | . | . | . |
| 61 | JX293001 | *Garra surendranathanii* | . | . | . | . | . | . | . | . | . | . | . | . | . | . | . | . | . | T | . | . | T | . | . | . | . | . | . | . | . | . | T | . | A | . | . | . | C | . | . | . |
| 62 | MK902678 | *Garra surinbinnani* | . | . | . | . | . | . | . | . | . | . | . | . | . | . | A | . | . | . | . | . | C | . | . | . | . | . | . | . | . | . | . | . | . | . | . | . | . | . | . | . |
| 63 | KX244651 | *Garra sindhi* | . | . | A | . | . | . | . | . | G | . | . | . | . | . | A | . | . | . | . | . | C | . | . | . | . | . | . | . | . | T | . | . | . | T | . | . | C | . | . | C |
| 64 | KY365751 | *Garra tashanensis* | . | . | . | . | . | . | . | . | . | . | . | . | . | . | A | . | . | . | . | . | C | . | . | . | . | . | . | . | . | T | . | . | A | T | . | . | . | . | . | C |
| 65 | JQ864607 | *Garra tengchongensis* | . | . | . | . | . | . | . | . | . | . | . | . | . | . | A | . | . | A | . | . | C | . | . | C | . | . | . | . | . | . | . | . | A | T | . | . | . | . | . | C |
| 66 | ON072400 | *Garra* cf. *trewavasae* | . | . | . | . | . | . | . | . | . | . | . | . | . | . | A | . | . | T | . | . | C | . | . | . | . | . | . | . | . | T | . | . | A | . | . | . | C | . | . | C |
| 67 | MH716242 | *Garra turcica* | . | . | . | . | . | . | . | . | G | . | . | T | . | . | A | . | . | T | . | . | C | . | . | . | . | . | . | . | . | T | . | . | A | T | . | . | . | . | . | . |
| 68 | KM214731 | *Garra typhlops* | . | . | . | . | . | . | . | . | . | . | . | T | . | . | A | . | . | T | . | . | C | . | . | . | . | . | . | . | . | T | . | . | A | . | . | . | C | . | G | . |
| 69 | KM214800 | *Garra variabilis* | . | . | . | . | . | . | . | . | G | . | . | T | . | . | A | . | . | T | . | . | C | . | . | . | . | . | . | . | . | T | . | . | A | T | . | . | . | . | . | C |
| 70 | MK572214 | *Garra vittatula* | . | . | C | . | . | . | . | . | . | . | . | T | . | . | A | . | . | A | . | . | . | . | . | . | . | . | C | . | . | . | T | . | A | . | . | . | . | . | . | C |
| 71 | MN167177 | *Garra vinciguerrae* | . | . | A | . | . | . | . | . | . | . | . | . | . | . | A | . | . | T | . | . | C | . | . | . | . | . | . | . | . | . | . | . | A | T | . | . | C | . | . | C |

*Species that found in Mekong River
